# Correction: Castro et al. Stakeholders’ Understanding of European Medicine Agency’s COVID-19 Vaccine Information Materials in EU and Regional Contexts. *Vaccines* 2023, *11*, 1616

**DOI:** 10.3390/vaccines13101049

**Published:** 2025-10-13

**Authors:** Indiana Castro, Marie Van Tricht, Nicole Bonaccorso, Martina Sciortino, Juan Garcia Burgos, Claudio Costantino, Rosa Gonzalez-Quevedo

**Affiliations:** 1 Public and Stakeholder Engagement Department, European Medicines Agency, Domenico Scarlattilaan 6, 1083 HS Amsterdam, The Netherlands; 2 Department of Health Promotion Sciences, Maternal and Infant Care, Internal Medicine and Medical Specialties (PROMISE) “G. D’Alessandro”, University of Palermo, 90127 Palermo, Italy; martina.sciortino@unipa.it (M.S.); claudio.costantino01@unipa.it (C.C.)

The author would like to make the following correction to this publication [1]. 

In the original publication, the mandatory disclaimer required for all scientific publications authored by European Medicines Agency (EMA) staff was inadvertently omitted. The correct disclaimer is provided below and should be included immediately after the Conflict of Interest section:

“Disclaimer: The views expressed in this article are the personal views of the authors and may not be understood or quoted as being made on behalf of or reflecting the position of the regulatory agency/agencies or organisations with which the authors are employed/affiliated.”

The authors state that the scientific conclusions are unaffected. This correction was approved by the Academic Editor. The original publication has also been updated.
