# Supplementary material for: Stakeholders’ Understanding of European Medicine Agency’s COVID-19 Vaccine Information Materials in EU and Regional Contexts
Source: Vaccines (Basel). 2023 Oct 19;11(10):1616. doi: 10.3390/vaccines11101616 (PMC10610863; doi:10.3390/vaccines11101616)
Supplement: Supplementary file 1 [file vaccines-11-01616-s001.zip › Supplementary Material S1.pdf]

EUROPEAN MEDICINES AGENCY
SCIENCE MEDICINES HEALTH

4 September 2020

COVID-19 vaccines in the EU

Basic facts

Why are vaccines to prevent COVID-19 urgently needed?

- COVID-19 vaccines are medicines that aim to prevent disease caused by the novel coronavirus SARS-CoV-2 by triggering an immune response.
- The current COVID-19 pandemic is a global crisis, with devastating health, social and economic impact.
- COVID-19 can cause severe disease and death with yet unknown long-term consequences in people of all ages, including in otherwise healthy people.
- Safe and effective vaccines for COVID-19 are needed to protect individuals from becoming ill, especially healthcare professionals and vulnerable populations, such as older people or those with chronic diseases.

Is there a vaccine to protect against COVID-19?

- There are no vaccines approved yet in the EU to prevent or treat any of the human coronavirus infections, including those causing the common colds or more serious conditions.
- Due to the urgency posed by the pandemic, efforts are ongoing to develop and study COVID-19 vaccines in order to approve and make them available as soon as possible.
- It is not currently known what level of protection can be reached with the vaccines in development. Reasonably effective vaccines, together with other public health measures and therapeutic treatments, will be a key component in overcoming COVID-19.
- Vaccines work by preparing a person's immune system (the body's natural defences) to recognise and defend itself against a specific disease. Most research on COVID-19 vaccines involves generating responses to all or part of a protein (spike protein, or protein S) that is unique to the virus that causes COVID-19. When a person receives the vaccine, it will trigger an immune response. If the person is infected by the virus later on, the immune system recognises the virus and, because it is already prepared to attack the virus, protects the person from COVID-19.

What process and methods are being used to develop and approve COVID-19 vaccines?

- COVID-19 vaccines are being developed following the same legal requirements for pharmaceutical quality, safety and efficacy as other medicines.
- Like all medicines, COVID-19 vaccines' effects are first tested in laboratory, including in animals, and then vaccines are tested in human volunteers.
- Before approval, all vaccines in the EU are evaluated against the same high standards as any other medicine.
- What is different for COVID-19 vaccines is that speed of development and potential approval is much faster due to the public health emergency.
- EMA has put in place a dedicated expert task force and rapid review procedures to evaluate high-quality applications from companies in the shortest possible timeframes, while ensuring robust scientific opinions.
- The European Commission will make use of all existing flexibilities to accelerate the approval of any potential vaccines for use across the EU, but this will only be possible if EMA receives sound scientific evidence that allows establishing that vaccines' benefits are greater than any risks.
- Vaccine manufacturers and academics are using established production systems currently used for safe and effective vaccines. In addition, they are continuously researching novel approaches to producing and developing vaccines, and some of the advances made to date are also being applied to developing vaccines for COVID-19.
- Some vaccines for COVID-19 are being developed using novel methods that are expected to increase the volume and speed of production compared to other types of vaccines, enhance product stability and bring about strong immune responses.
- Other vaccines are being developed using existing methods. These are already used in vaccines for other diseases, which means it could be easier to use existing production facilities to produce COVID-19 vaccines at a large scale than for newer vaccine types.

Why did development only start after the pandemic was declared?

- Vaccines can only be developed when the infectious agent is known. Because SARS-CoV-2 is a new virus that had not been seen before, development of a vaccine to protect against COVID-19 could only be started once the virus emerged and its genetic make-up had been analysed. However, vaccine development builds on experience and technologies used for other vaccines.

When will the vaccines be approved?

- At present it is not known whether COVID-19 vaccines will be approved or how long this will take, as timelines are difficult to predict.
- Vaccine development is progressing, and, in a best-case scenario, the Agency would receive clinical data on the most advanced vaccines in development towards the end of 2020.
- Regulators would then carry out a scientific evaluation of the vaccine's safety, efficacy and quality, before concluding on whether there is sound scientific evidence supporting approval.

- If the scientific evidence supports a positive benefit-risk assessment for any of the vaccines, the European Commission will grant a marketing authorisation valid across the EU in the shortest possible timelines.
- For evaluation of other vaccines currently at earlier development stages, this would likely take place throughout 2021 and beyond.

What type and amount of data is needed for approving a safe and effective vaccine?

- COVID-19 vaccine developers need to submit specific data on their vaccine. EMA then carries out a thorough assessment of these data to reach a scientific opinion on whether the vaccine is safe, efficacious and of good quality and is therefore suitable to vaccinate people.
- The data should show the vaccine's efficacy in protecting against COVID-19 (how well the vaccine works in clinical settings) and its safety.
- Efficacy is measured by looking at how well the vaccine works in the study, for example how well the vaccine prevents symptomatic disease. These efficacy measures are called 'endpoints'. Efficacy endpoints are required because COVID-19 is a new disease and because there are no known indicators (such as the levels of antibodies in the blood) that can predict protection.
- The safety requirements for COVID-19 vaccines are the same as for any other vaccine in the EU and will not be lowered in the context of the pandemic.
- The data submitted in a marketing authorisation application for a COVID-19 vaccine must include information on:
  - the group of people to be given the vaccine;
  - its pharmaceutical quality, including information on the identity and purity of the vaccine components and its content and biological activity (potency);
  - data on each step of manufacturing and on the controls used to ensure that each batch of vaccine is consistently of good quality;
  - compliance with international requirements for laboratory testing, vaccine manufacture and conduct of clinical trials ('good laboratory practice', 'good clinical practice' and 'good manufacturing practice');
  - types of immune responses induced by the vaccine;
  - the effects observed in the groups of people to be given the vaccine;
  - the vaccine's side effects observed in vaccinees, including if there are any data in special populations such as older people or pregnant women;
  - information intended to be gathered from follow-up studies after authorisation (e.g. long-term safety data or long-term immunity);
  - prescribing information to be provided to patients and healthcare professionals (i.e. the summary or product characteristics or SmPC, labelling and package leaflet), which is drafted by the developer and reviewed and agreed by EMA's scientific committees;
  - the way risks will be managed and monitored once the vaccine is authorised; the risk management plan (RMP), a document with information about any possible (known or potential) safety concerns with the vaccine, the way risks will be managed and monitored

once the vaccine is authorised and what information is intended to be gathered from follow-up studies. The RMP is evaluated by EMA's safety committee, PRAC.

How long will immunity from a vaccine last?

- Currently, because the virus is so novel, there is not enough knowledge on how long the immunity conferred by the vaccines will last after vaccination, or whether there will be a need for periodic booster doses.
- Data from immunogenicity and efficacy studies in the long term will inform future vaccination strategies.
- Vaccination policies are not decided by EMA but by public health agencies in EU member states.

Will vaccines protect vaccinated people if the virus mutates?

- Typically, viruses mutate (the genetic material in the virus changes); this happens at different rates for different viruses and mutations do not necessarily affect how well the vaccine works against the virus.
- Some vaccines against viral diseases remain effective many years after their development and provide long-lasting protection, such as vaccines for measles or rubella.
- On the other hand, for diseases such as flu, virus strains change so often and to such an extent that the vaccine composition must be updated on a yearly basis for it to be effective.
- The scientific community and regulators will monitor whether the coronavirus Sars-CoV-2 changes over time and, if so, whether vaccines can protect people from infection with new variants.

20 November 2020
EMA/519954/2020

COVID-19 vaccines in the EU

Development, scientific evaluation, approval and monitoring

The European Medicines Agency (EMA) plays an important role in enabling the development, scientific evaluation, approval and monitoring of COVID-19 vaccines in the European Union (EU).

I. Development of COVID-19 vaccines

- COVID-19 vaccines are being developed according to current regulatory guidelines and legal requirements. Like all medicines (Figure 1), COVID-19 vaccines are first tested in the laboratory (e.g. studies on their pharmaceutical quality and studies to check first the effects in laboratory tests and animals). Then vaccines are tested in human volunteers in studies called clinical trials. These tests help confirm how the vaccines work and, importantly, to evaluate their safety and protective efficacy.

Figure 1: Overview of vaccine development and approval stages

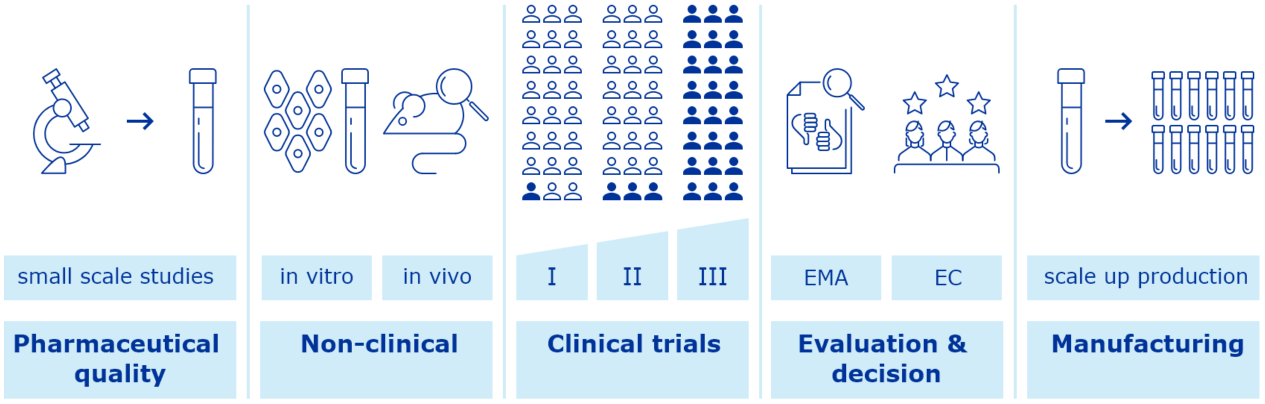

Standard vaccine development

- Standard vaccine development is a long process (Figure 2a) and studies are done in sequential steps; companies first make small batches and do small scale studies to characterise/optimise the production process. Studies are performed to determine a suitable formulation that can keep vaccine components stable to the end of its shelf life. Then the company decides whether to continue development and scale up production. A suitable and effective quality control strategy is developed to assure that the vaccine meets its intended quality profile and complies with regulatory standards.
- Studies on pharmaceutical quality look at the individual vaccine components, the final formulation to be used and at the whole manufacturing process in detail.
- More studies are done in laboratory models, using in vitro studies or animal models (in vivo studies), to show how the vaccine triggers an immune response and works to prevent infection.
- Finally, the vaccine developer studies the vaccine in three phases of clinical trials, with larger numbers of volunteers in each phase:
    - ***Human pharmacology studies (or Phase I trials)*** generally study between 20 - 100 healthy volunteers to confirm that the medicine behaves as expected from laboratory tests. For instance: Does the vaccine trigger the expected immune response? Is the vaccine safe to move into larger studies? Which doses can be adequate?
    - ***Therapeutic exploratory studies (or Phase II trials)*** are done in several hundred volunteers and study the best doses to use, the most common side effects and how many doses are needed. These studies also check that the vaccine triggers a good immune response in a broader population. In certain cases, it could also provide some preliminary indications of how well the vaccine will work (efficacy).
    - ***Clinical efficacy and safety studies (Phase III trials)*** include thousands of volunteers and show how efficacious the vaccine is at protecting against the infection compared with placebo (dummy) or alternative treatment and what are the less common side effects in those receiving the investigational vaccine. Measures of how efficacious the vaccine is could be reduction in the number of people with symptoms, reduction in the number of people with severe disease or reduction in the number of people diagnosed with the infection.
- Clinical trials in the EU, including those for COVID-19 vaccines, are authorised and managed at national level. National competent authorities and ethics committees ensure that studies are scientifically sound and conducted in an ethical manner.

Figure 2: Indicative timelines for COVID-19 vaccines compared with standard vaccines

2a. Standard vaccines

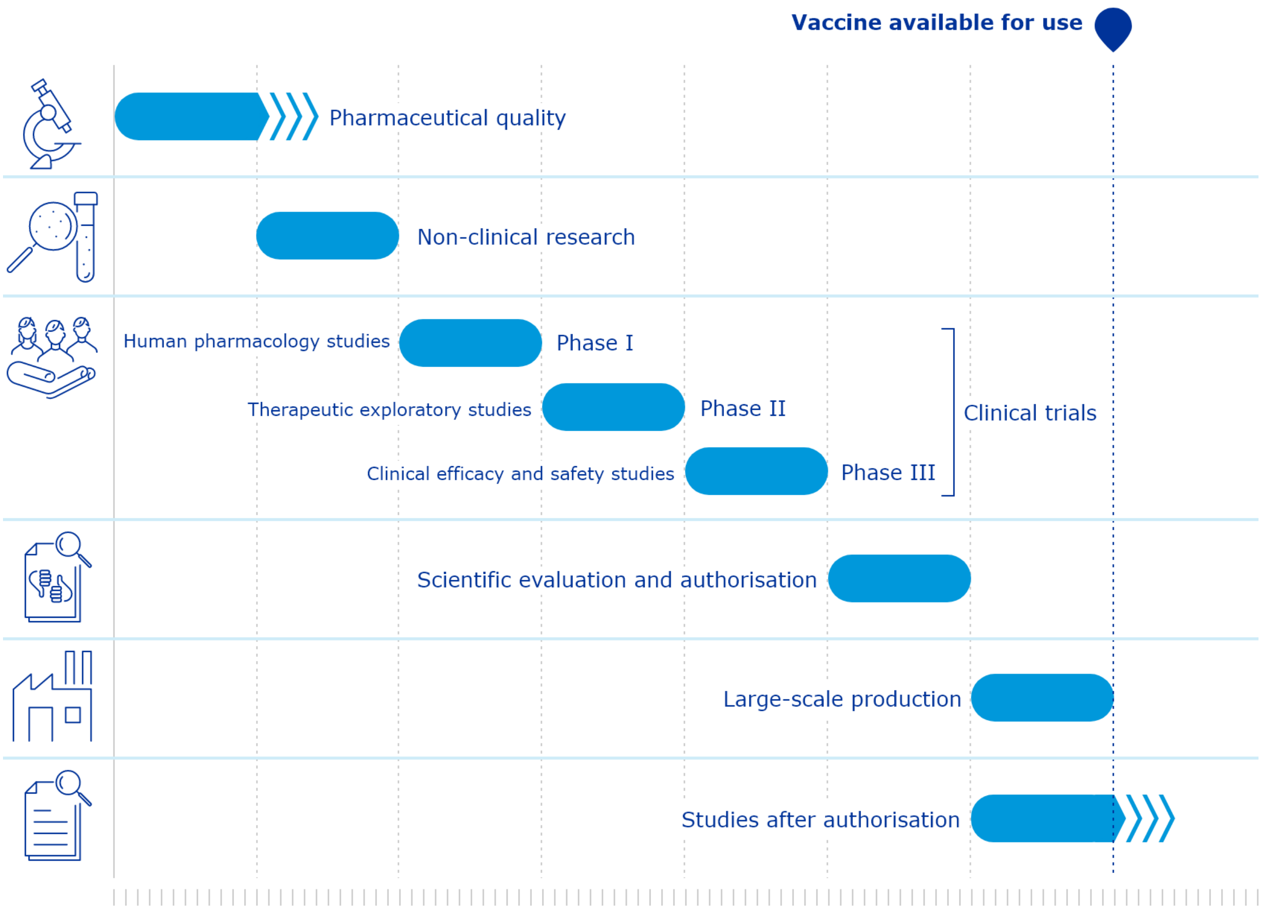

Fast-track vaccine development in a public health emergency

- Due to the public health emergency, vaccine development for COVID-19 vaccines is being fast-tracked globally (Figure 2b).
- This implies that development is compressed in time, applying the extensive knowledge on vaccine production gained with existing vaccines. Companies may use different approaches to reduce timelines, for example:
  - mobilise more extensive human resources simultaneously, allowing them to analyse results from earlier studies more quickly and map out next steps in terms of resources, funding and regulatory strategy;
  - combine clinical trial phases or conduct some studies in parallel, instead of carrying them out sequentially, where it is safe to do so;

2b. COVID-19 vaccines

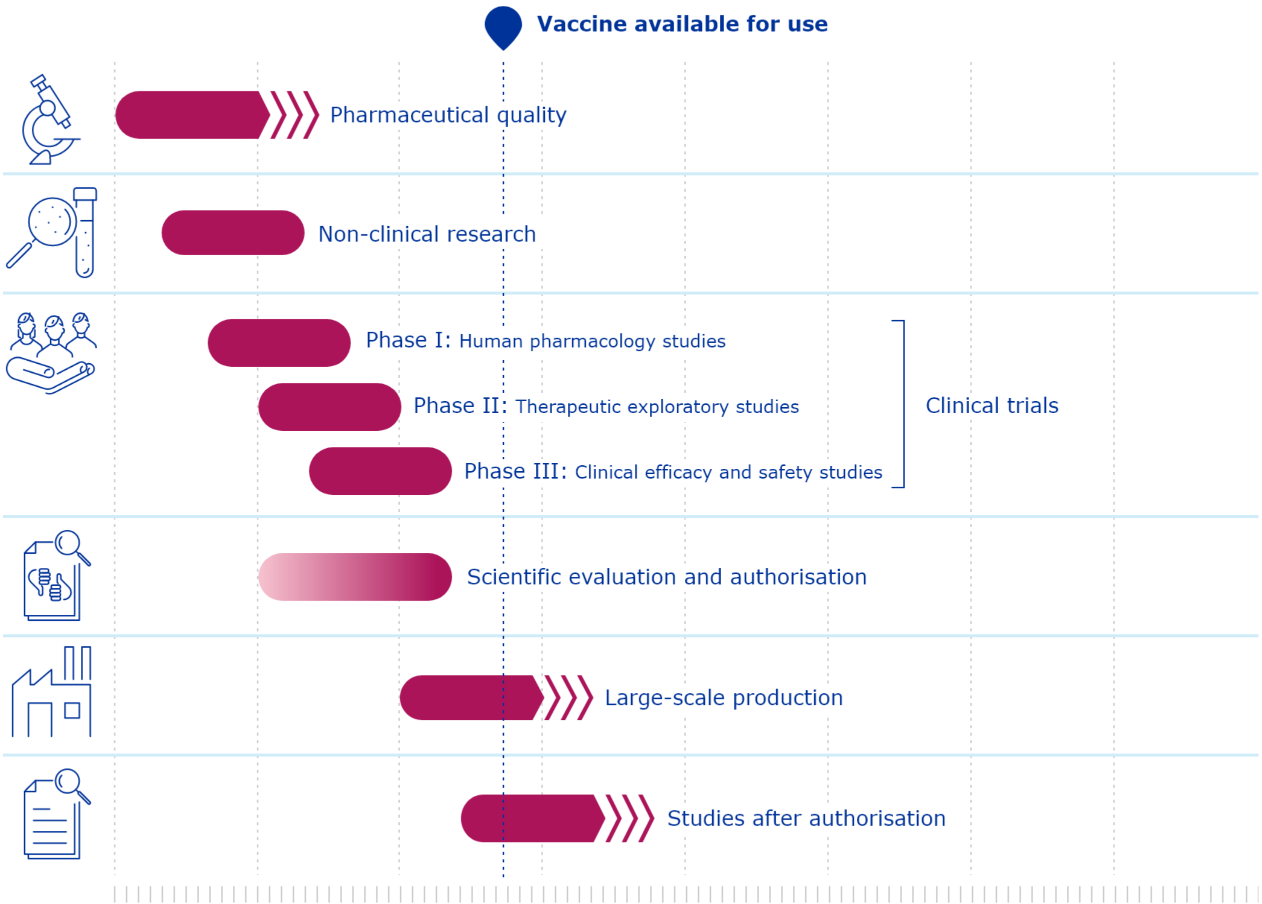

- Early scientific advice from regulators also helps speed up development:
  - In the case of COVID-19 vaccines the Agency is having early and continuous dialogue with companies whose vaccines are considered of high public health priority, so that they can receive and implement any regulatory advice on planned studies as they go along, to ensure studies are well designed and meet regulatory criteria.
  - In the EU, for each COVID-19 vaccine under development, early dialogue takes place between EMA and individual companies to discuss the strategy for evidence-generation. This is done through EMA's COVID-19 Task Force (ETF) and its scientific advice process.
  - Advising companies on regulatory requirements helps to ensure that standards of quality, safety and efficacy are embedded early in the process and are not compromised by fast-track development. COVID-19 vaccines can only be approved and used if they comply with all the requirements of quality, safety and efficacy set out in the EU pharmaceutical legislation.
  - EMA is offering rapid scientific advice to vaccine developers so they can receive prompt guidance and direction on the best methods and study designs to generate robust data on how well a medicine or vaccine works, how safe it is, as well as on the manufacturing and control process to establish its quality.
- In parallel to fast tracking development, companies are expanding manufacturing capacity and large-scale production, to facilitate vaccine deployment without further delay once approved. In

the EU, this is further encouraged because the European Commission has provided support to facilitate vaccine development and deployment as quickly as possible: https://ec.europa.eu/info/sites/info/files/communication-eu-strategy-vaccines-covid19_en.pdf

- The Agency is not involved in providing incentives to vaccine developers working on COVID-19 vaccines other than enabling access to regulatory procedures, (e.g. free scientific advice during vaccine development: https://www.ema.europa.eu/en/news/covid-19-developers-medicines-vaccines-benefit-free-scientific-advice). In addition, EMA has no role in negotiating potential vaccine availability, funding and deployment at EU or national level.

Figure 3: Key components of vaccine development - standard vaccines compared with COVID-19 vaccines

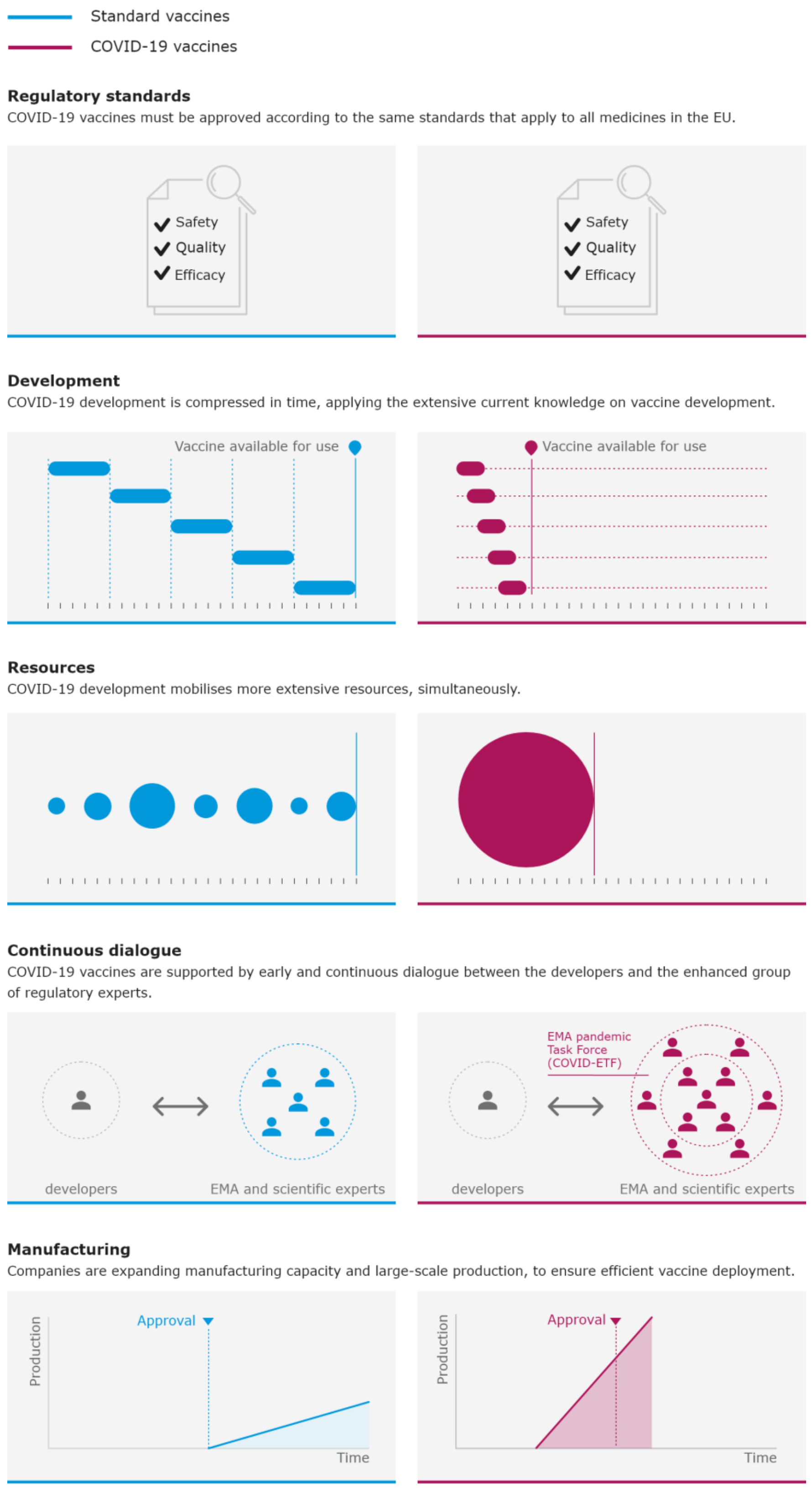

II. Scientific evaluation and approval process for COVID-19 vaccines

- COVID-19 vaccines must be approved according to the same standards of pharmaceutical quality, safety and efficacy that apply to all medicines in the EU. These standards are reflected in the EU pharmaceutical law, which all companies developing vaccines must follow, and they will not be lowered in the pandemic context.

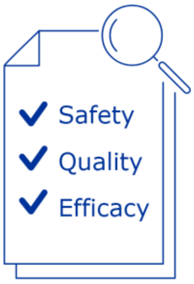

- In view of the pandemic, EMA and regulatory agencies in Europe are diverting resources to speed up processes and reduce timelines for the evaluation and authorisation of COVID-19 vaccines.

Robust regulatory framework and scientific expertise in the EU

- The EU's robust pharmaceutical legislation ensures that vaccines are only approved after scientific evaluation of their quality, safety and efficacy has demonstrated that their overall benefits outweigh their risks (Figure 4).
- For COVID-19 vaccines, this evaluation needs to show that a vaccine's benefits in protecting people against COVID-19 are far greater than any side effect or potential risks.

Figure 4. Vaccines' benefits outweigh their side effects or potential risks

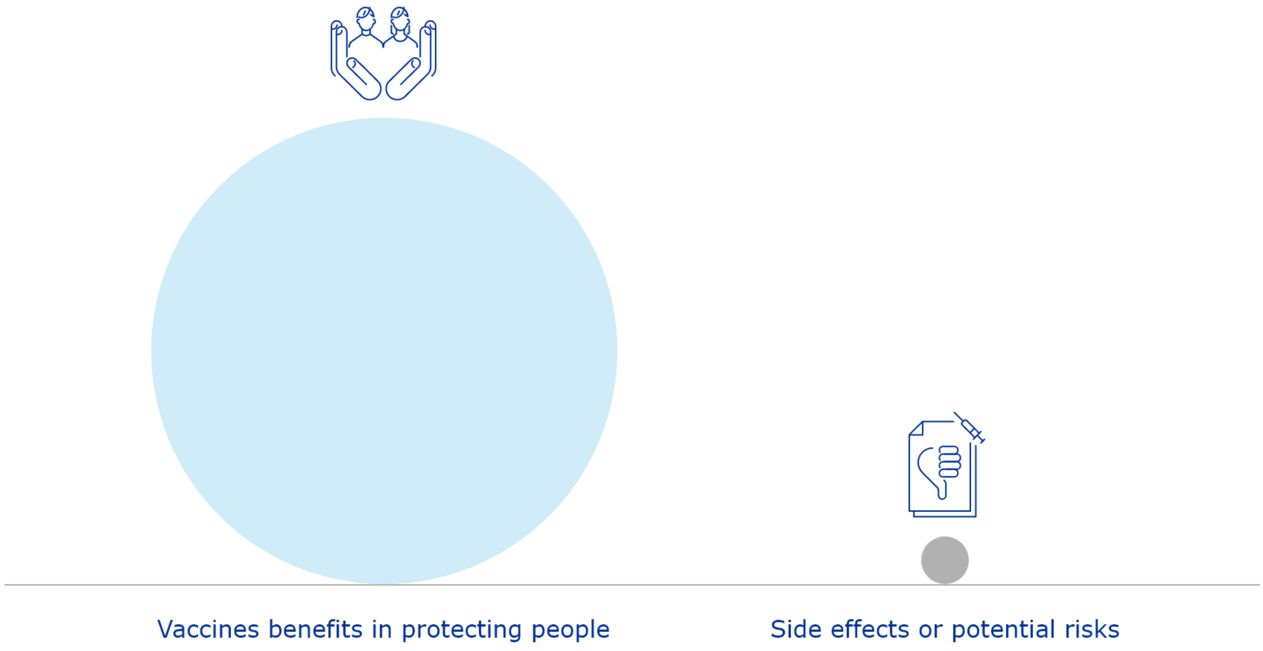

- The Agency (EMA) takes care to ensure that scientific experts evaluating medicines do not have any financial or other interests that could affect their impartiality. This is done by applying restrictions if competing interests are considered to potentially impact the impartiality of individual experts: https://www.ema.europa.eu/en/about-us/how-we-work/handling-competing-interests

- Independence of EMA's scientific evaluations is safeguarded also by a high level of transparency, which opens EMA's scientific evaluation work to public scrutiny. For COVID-19 medicines, the Agency will apply the highest level of transparency ever provided for medicines. Exceptional transparency measures have been put in place to meet an unprecedented public demand for information, support and make global research more efficient and allow public scrutiny and independent review.

Scientific evaluation and approval processes

- To gain marketing approval for a vaccine in the EU (Figure 5), the vaccine developer submits the results of all testing/investigations to the medicines regulatory authorities in Europe as part of a 'marketing authorisation' application.

- Most COVID-19 vaccines in the EU will be evaluated by EMA (via the 'centralised procedure'). Any vaccine produced using biotechnology will be evaluated by this route.

- EMA's evaluations are carried out by its expert scientific committees on human medicines (the CHMP and PRAC), made up of experts working in national medicines' regulatory agencies.

- As for all medicines, EU law requires that the initial evaluations are carried out separately by two different teams (Rapporteur and Co-Rapporteur) and reviewed by EMA's human medicines committee (CHMP) as a whole, to ensure a balanced view. For more information see: https://www.ema.europa.eu/en/from-lab-to-patient-timeline.

- To streamline and focus the work of the Committees and related Working Parties on medicines for the pandemic, EMA has set up a multi-disciplinary COVID-19 specific working group, the COVID-19 Task Force (ETF) bringing together key experts of the European medicines regulatory network, including with experts on infectious diseases, clinical trials and vaccines safety and manufacture, to ensure a fast and coordinated response to the COVID-19 pandemic.

- The review by EMA results in a scientific opinion which is then sent to the EC, which ultimately grants an EU-wide marketing authorisation in case of a positive outcome.

- As part of the approval process, regulators may carry out inspections to further assure that the information the vaccine developer provides has been quality assured and generated in strict compliance with regulatory standards, and that the studies have been carried out as described. This is always done for new manufacturing sites.

- Only after regulatory approval and thorough quality control, can a COVID-19 vaccine be introduced into national healthcare systems and used to protect people. The details for such national processes may be different for each member state.

Figure 5. Evaluation and approval steps for COVID-19 vaccines

Approval steps:	Who is involved:	
Marketing authorisation application	**Vaccine developers**	Submit the results of all testing to the medicines regulatory authorities in Europe
EMA's evaluation and scientific opinion	**EMA scientific experts (CHMP, PRAC)**	Carry out scientific evaluation of vaccines
	**EMA pandemic Task Force (COVID-ETF)**	Enables EU Member States and the European Commission to take quick and coordinated regulatory action on the development, authorisation and safety monitoring of treatments and vaccines intended for the treatment and prevention of COVID-19
European Commission review and authorisation	**European Commission**	Reviews EMA's scientific opinion and grants an EU-wide marketing authorisation in case of a positive outcome
Vaccine adoption in national healthcare systems	**National authorities**	Decide on introduction of the newly approved vaccine and vaccination policies

- Each batch of vaccine released onto the EU market is always tested prior to release. Stringent testing is done by the company holding the marketing authorisation and batches must meet the corresponding specifications approved by authorities.
- For vaccines to be used in public health immunisation programmes, such as those that may be approved via EMA for COVID-19, an additional independent control for each batch of vaccine, performed by an official medicines control laboratory (OMCL), is normally required before the company can market the respective batch: https://www.edqm.eu/en/batch-release-human-biologicals-vaccines-blood-and-plasma-derivatives.
- This independent control is referred to as Official Control Authority Batch Release (OCABR) and includes testing of agreed quality parameters and a careful review of the compliance of the manufacturer's own test results.

Accelerated evaluation for approval

- The EU legislation and procedures provide several tools that can be used in the event of an emerging threat or outbreak, mainly to expedite evaluation of potential vaccines.
- According to the EU pharmaceutical legislation, the standard timeline for the evaluation of a medicine is a maximum of 210 active days. However, for COVID-19 medicines, EMA is applying an expedited procedure called rolling review.

Rolling review

- ✓ This procedure (Figure 6), used in a public health emergency, allows EMA to assess data for a promising medicine as soon as they become available on a rolling basis.
- ✓ In normal circumstances, all data supporting a marketing authorisation application must be submitted at the start of the evaluation procedure. In the case of a rolling review, leads for the evaluation (rapporteurs) are appointed whilst development is still ongoing and the Agency reviews data as they become available.
- ✓ Several rolling review cycles can be carried out during the evaluation of a vaccine as data continue to emerge, with each cycle lasting at least two weeks, depending on the amount of data to be assessed.
- ✓ Once the data package is considered complete, a developer can submit a formal marketing authorisation application to EMA, which can then be processed very quickly, because most of the data have already been reviewed during the rolling process.
- ✓ How long a rolling review will take can be difficult to predict, as it will depend on many factors such as the robustness of data presented by the company.

Figure 6: Standard evaluation process compared with rolling review of COVID-19 vaccines

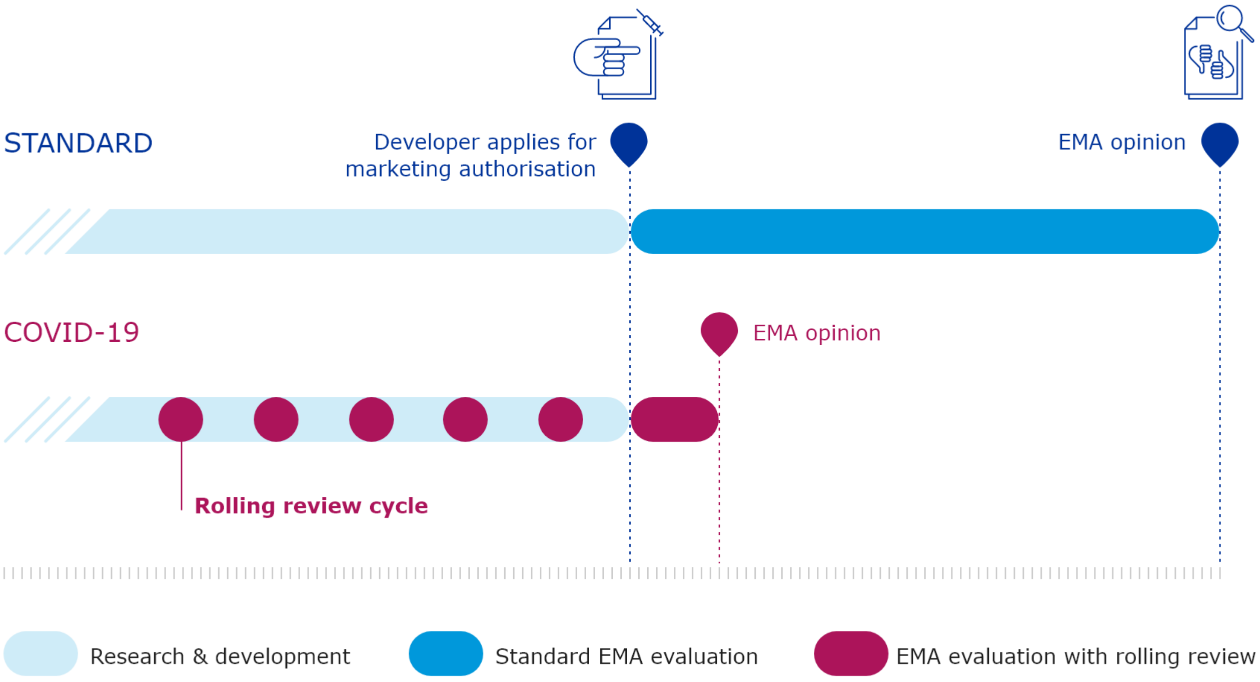

- When an evaluation is complete, EMA has the option of recommending a **conditional marketing authorisation**, a type of approval for medicines addressing unmet medical needs, and in particular those to be used in emergency situations in response to public health threats recognised by the WHO or the EU:

✓ This type of approval can be granted if, despite the available data being not as extensive as normally required for an approval, the benefits of faster access to a potentially life-saving medicine outweigh the risks of having less comprehensive data. Approval can then be granted on the condition that the company will supply the additional complementary information within defined timelines, including the results of further studies, once the vaccine is on the market.

✓ The data required for a conditional marketing authorisation for a vaccine will vary case by case. Often in an emergency situation the data that need to be supplied after marketing will be from clinical studies as well from observational studies investigating effectiveness and safety.

✓ Some pharmaceutical (quality) or non-clinical data (from studies in animals or in a laboratory) may be provided later as well, for example, studies on long term stability and for how long animals are immune can be provided later, while other key studies to prove quality and safety must be provided initially.

✓ What data is essential before approval (e.g. during rolling review) and what can be provided afterwards will depend on the individual vaccine and its balance of benefits and risks, taking into account factors such as what is known about that type of vaccine.

✓ Where it is agreed that specific pharmaceutical, clinical or non-clinical data may be provided later on, the data that is provided must still sufficiently support a positive benefit/ risk evaluation.

III. Monitoring safety and how well COVID-19 vaccines work in real life

- Like any medicine, vaccines have benefits and risks, and although highly effective, no vaccine is 100 percent effective in preventing a disease or 100 percent safe in all vaccinated people.
- Because vaccines are given to otherwise healthy people, clinical data are needed to demonstrate that the benefits are far greater than any side effect or potential risks.
- The safety requirements for COVID-19 vaccines are the same as for any other vaccine in the EU and will not be lowered in the context of the pandemic.
- Before a vaccine is approved for use, the main body of evidence for its safety and efficacy (how well the vaccine works as measured in clinical trials) comes from the results of controlled randomised clinical trials, where participants are selected based on specific entry criteria, randomly allocated to vaccination and followed up under controlled conditions in line with strict protocols.
- After authorisation the vaccine will be used in a larger number of people ('real-life' patients). Certain side effects, particularly rare or very rare ones, may only emerge, for example, when millions of people are vaccinated. EU law therefore requires that the safety of all medicines, including vaccines, is monitored while they are in use.
- How well the vaccine works in these real-life settings, for example at a wider population level, is called 'effectiveness'. Studies collecting effectiveness data would give additional information, for example, on long term protection or on the need for and timing of booster doses, to complement the 'efficacy' data obtained in clinical trials.

Standard monitoring

- The EU has a comprehensive safety monitoring and risk management (pharmacovigilance) system, which ensures measures are in place for:
    - providing advice to minimise risk;
    - reporting suspected side effects;
    - conducting studies after marketing;
    - detecting any potential adverse effects;
    - conducting rigorous scientific assessments of all safety data;
    - introducing any necessary mitigating actions early on.
- Competent authorities carry out safety and efficacy studies after marketing and can require a company to carry out studies as an obligation of the authorisation. Public authorities responsible for vaccination programmes will conduct other studies.
- Studies collecting effectiveness data give additional information, for example, on long term protection or on the need for and timing of booster doses, to complement the efficacy data.

Large scale monitoring activities in the pandemic context

- Regulators and vaccine developers are mobilising extra resources to monitor safety and manage risk in the pandemic. Although large numbers of people will receive COVID-19 vaccines in clinical trials, this is important because exceptionally large numbers of people are expected to receive them once authorised.
- The pharmacovigilance plan for COVID-19 vaccines sets out how EMA and the national competent authorities in the EU Member States identify and evaluate any new information that arises promptly, including any safety signals that are relevant for the benefit-risk balance of these vaccine: https://www.ema.europa.eu/en/documents/other/pharmacovigilance-plan-eu-regulatory-network-covid-19-vaccines_en.pdf
- This plan also ensures that regulators can take any appropriate regulatory actions and communicate these to the public as quickly as possible.
- The monitoring activities in the plan apply to all vaccines, but they take place on a larger scale during this pandemic:
    - Collecting exposure data to COVID-19 vaccines
    - Adopting specific safety signal detection and management measures
    - Setting up a European infrastructure for monitoring COVID-19 treatments and vaccines
    - Using real-world data from clinical practice
    - Applying exceptional transparency measures
- EMA's guidance on preparing risk management plans for COVID-19 vaccines helps companies to develop risk management plans for COVID-19 vaccines: Guidance on risk management plans for COVID-19 vaccines.
- The risk management plan (RMP) is a document that details information about any possible (known or potential) safety concerns with the vaccine, the way risks will be managed and monitored once the vaccine is authorised and what information is intended to be gathered from follow-up studies.

The RMP plans sets out how the company will monitor and report on safety and how it will characterise and manage risks following authorisation of a COVID-19 vaccine and is evaluated by EMA's safety committee, PRAC.

- Companies need to submit monthly safety reporting summaries for COVID-19 vaccines, in addition to periodic safety update reports, and put processes in place to manage a high volume of safety reports. They need to carry out further studies on COVID-19 vaccines that receive a conditional marketing authorisation.
- Additional considerations in this guidance address traceability tools that can help record who has received which vaccine and from which batch.
- EMA publishes the full body of the risk management plans (plus Annex 4) for all authorised COVID-19 vaccines, in line with its exceptional transparency measures for COVID-19 medicines: https://www.ema.europa.eu/en/human-regulatory/overview/public-health-threats/coronavirus-disease-covid-19/treatments-vaccines/transparency-exceptional-measures-covid-19-medicines
