# Supplementary material for: Stakeholders’ Understanding of European Medicine Agency’s COVID-19 Vaccine Information Materials in EU and Regional Contexts
Source: Vaccines (Basel). 2023 Oct 19;11(10):1616. doi: 10.3390/vaccines11101616 (PMC10610863; doi:10.3390/vaccines11101616)
Supplement: Supplementary file 1 [file vaccines-11-01616-s001.zip › Supplementary Material S2.pdf]

Introduction

This user-testing evaluates two documents prepared by EMA on COVID-19 vaccines:

1. "COVID-19 vaccines: key facts"
2. "COVID-19 vaccines: development, evaluation, approval and monitoring"

Your feedback to this survey will help us check if these communication materials are clear and useful. It will help us improve how we explain COVID-19 vaccines to the public.

Please complete the survey only after you have read the two documents (attached in the email).

Please, first read the document 1 entitled "COVID-19 vaccines: key facts" and complete the first part of the survey.

The second document "COVID-19 vaccines: development, evaluation, approval and monitoring", is more detailed. Reading the second document and completing the second part of the survey is optional for individual patients.

This survey does not collect personal data, therefore you do not need to reveal your identity or include other personal data in the free text answers. The survey will collect the answers only in an aggregated and anonymous format. The results will also be shared in aggregated form and will not reveal individual responses. Regarding the processing of your data by the EUSurvey application, please refer to the specific privacy statement of the EUSurvey tool: https://ec.europa.eu/eusurvey/home/privacystatement

Background information

* Q1. Are you a (select 1-2 options):

- [ ] Individual patient
- [ ] Individual healthcare professional
- [ ] Patient Organisations member
- [ ] Healthcare Professional Organisations member
- [ ] Consumer
- [ ] Other

If other, please specify:

Q2. Before reading EMA's COVID-19 vaccines materials, how willing are you to get vaccinated against COVID-19 once a vaccine becomes available?

1=very unlikely. 2= unlikely. 3. Somewhat unlikely. 4= neither unlikely nor likely. 5= Somewhat likely. 6= likely. 7= very likely.

	1	2	3	4	5	6	7
1= very unlikely. 7=very likely.	○	○	○	○	○	○	○

Information sources and content format

* Q3. Where would you normally look for information regarding COVID-19 vaccines? (select all that apply)

☐ Your family doctor

☐ A pharmacist

☐ A nurse

☐ Your family, colleagues and/or friends

☐ Your community leader

☐ Social media (Facebook, Instagram, Twitter, Reddit, etc.)

☐ Internet search engine (Google search, Bing, etc.)

☐ Newspaper (printed or online)

☐ Scientific journals

☐ World Health Organization (WHO)

☐ European Medicines Agency (EMA)

☐ European Centre for Disease Prevention and Control (ECDC)

☐ Your country's Medicines Regulator Agency

☐ Your country's Ministry of Health

☐ Do not know where to look

☐ Other

If other, please specify:

* Q4. Is English your native language?

◯ Yes

◯ No

* Q5. If answered no, was the language used an obstacle for understanding the document(s)?

◯ Yes

◯ No

◯ I don't know

Feedback on EMA vaccines document 1: Key facts

The following questions apply to document 1 "COVID-19 vaccines: key facts". Please read this document before starting this section.

For the first 4 questions, please do not go back to check that your answer is correct. The survey is designed to collect the answers only in an aggregated and anonymous format. The purpose of these questions is not to assess your individual knowledge but to see if the document is easily understood. If you go and check the document again, it will introduce unwanted bias in our analysis.

* Q6. When will COVID-19 vaccines be approved in the EU?

◯ Before the end of 2020

◯ By May 2021

◯ By September 2021

Timelines are difficult to predict so it is unknown

* Q7. How does EMA ensure that COVID-19 vaccines will be safe and effective? (select all that apply)

- [ ] Skipping studies in human volunteers
- [ ] Reviewing study data faster but carefully
- [ ] With support from a dedicated expert task force
- [ ] Applying the same high standards as to any other medicine.
- [ ] Ensuring all studies are done correctly

* Q8. True or False: The safety requirements for COVID-19 vaccines are the same as for any other vaccine in the EU and will not be lowered in the context of the pandemic.

True

False

Q9. True or False: EMA carries out a scientific evaluation of the vaccine's safety, efficacy and quality, before concluding on whether the vaccine can be approved.

True

False

Q10. After finishing reading "COVID-19 vaccines key facts", did you find the information clear and helpful?

1= 1= very confusing and not helpful at all. 2= confusing and not helpful. 3= somewhat confusing but helpful. 4= neither confusing nor helpful. 5= somewhat clear and helpful. 6= clear and helpful. 7=very clear and helpful.

	1	2	3	4	5	6	7
*1= very confusing and not helpful at	○	○	○	○	○	○	○

	1	2	3	4	5	6	7
all. 7=very clear and helpful							

Q11. Could you now give a score to the following categories from 1 (not at all) to 7 (absolutely) according to your experience with the document?

1= not at all. 2= disagree. 3= somewhat disagree. 4= neutral. 5= somewhat agree. 6= agree. 7= absolutely.

	1	2	3	4	5	6	7
*Enough information has been provided to understand the key facts	○	○	○	○	○	○	○
*The language and vocabulary used in the document are easy to understand	○	○	○	○	○	○	○
*Did reading the document increased your understanding on COVID-19 vaccines?	○	○	○	○	○	○	○
*After reading "COVID-19 vaccines: key facts", how	○	○	○	○	○	○	○

	1	2	3	4	5	6	7
willing are you to get vaccinated against COVID-19 once a vaccine once becomes available? 1= very unlikely. 7=very likely.							

*Q12. Check the following link where the document "COVID-19 vaccines: key facts" is published as a webpage: https://www.ema.europa.eu/en/human-regulatory/overview/public-health-threats/coronavirus-disease-covid-19/treatments-vaccines/covid-19-vaccines-key-facts
Besides a webpage, would you find it useful to have this information in another shareable format?

- Yes
- No
- Don't know
- Other

If other, please specify:

Feedback on EMA vaccines document 2: Development, evaluation, approval and monitoring of COVID-19 vaccines

* The second document "COVID-19 vaccines: development, evaluation, approval and monitoring" is a more detailed document. If you are not a member of a patient or healthcare professional organisation, then reading the second document and completing the second part of the survey is optional.
If you have read it, please select "Yes" to continue with the survey.

- Yes
- No

For the first 3 questions, please do not go back to check that your answers are correct. The survey is designed to collect the answers only in an aggregated and anonymous format. The purpose of these questions is not to assess your individual knowledge but to check if the document is easily understood. If you go and check the document again, it will introduce unwanted bias in our analysis.

* Q13. When is vaccine safety monitored?

- Through all vaccine clinical development stages, including after approval has been granted and the vaccines are used in the population
- Only during phases 1-3 of human clinical trials
- Never
- Only once the vaccine is authorised

* Q14. What is a rolling review?

- The review done by the pharma companies producing the vaccines
- A review normally done by EMA when there are no pandemics going on
- A rapid and stringent review process that EMA is currently doing during the public health emergency to fast-track the evaluation of COVID-19 medicines

* Q15. Which of the below help with the development of COVID-19 vaccines?

- Combining some clinical trials phases, when safety is not compromised
- Early dialogue between developers and regulatory experts
- Using existing knowledge of safe and effective vaccines available on the market
- Expanding manufacturing capacity
- All the above are correct
- None of the above are correct

* Q16. The following statements are true regarding benefit-risk balance. Which statement do you think that captures more clearly that vaccines, as all medicines, have benefits and risks?

- Like any medicine, vaccines have benefits and risks, and although highly effective, no vaccine is 100% effective in preventing a disease or 100% safe in all vaccinated people.
- Like any medicine, vaccines have benefits and risks, and the conditions of marketing authorization aim at maximizing benefits and minimization risks.

Q17. After finishing reading "COVID-19 vaccines: Development, evaluation, approval and monitoring", did you find the information clear and helpful?

	1	2	3	4	5	6	7
*1=confusing and not helpful at all. 7= clear and helpful	○	○	○	○	○	○	○

Q18. Could you now give a score to the following categories from 1 (not at all) to 7 (absolutely) according to your experience with the document?

	1	2	3	4	5	6	7
*Enough information has been provided to understand the main messages	○	○	○	○	○	○	○
The graphics help to convey key concepts							
*The language and vocabulary used in the document are easy to understand	○	○	○	○	○	○	○

	1	2	3	4	5	6	7
*Did reading the document increased your understanding on COVID-19 vaccines?	◯	◯	◯	◯	◯	◯	◯
*After reading "COVID-19 vaccines: Development, evaluation, approval and monitoring", how likely are you to get vaccinated against COVID-19 once a vaccine once becomes available? 1= very unlikely. 7=very likely.	◯	◯	◯	◯	◯	◯	◯

* Q19. Please look over the two figures below for a half a minute. Do you think they are clear and easy to understand?

◯ Yes

◯ No

◯ I don't know

Resources figures

A

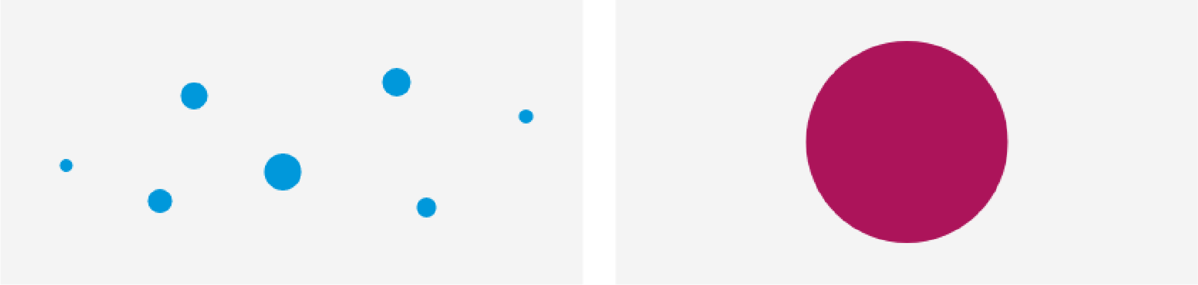

Resources

COVID-19 development mobilises more extensive resources, simultaneously.

B

Resources

COVID-19 v

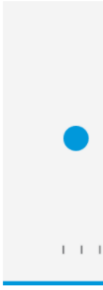

Q20. If answered yes, which of the figures do you prefer?

- A
- B

If you have any suggestion on any of the figures presented in the documents, please describe below:

* Q21. Please look over the three figures for a half a minute. Do you think they are clear and easy to understand?

- Yes
- No
- I don't know

Benefit-risk balance figures

A

B

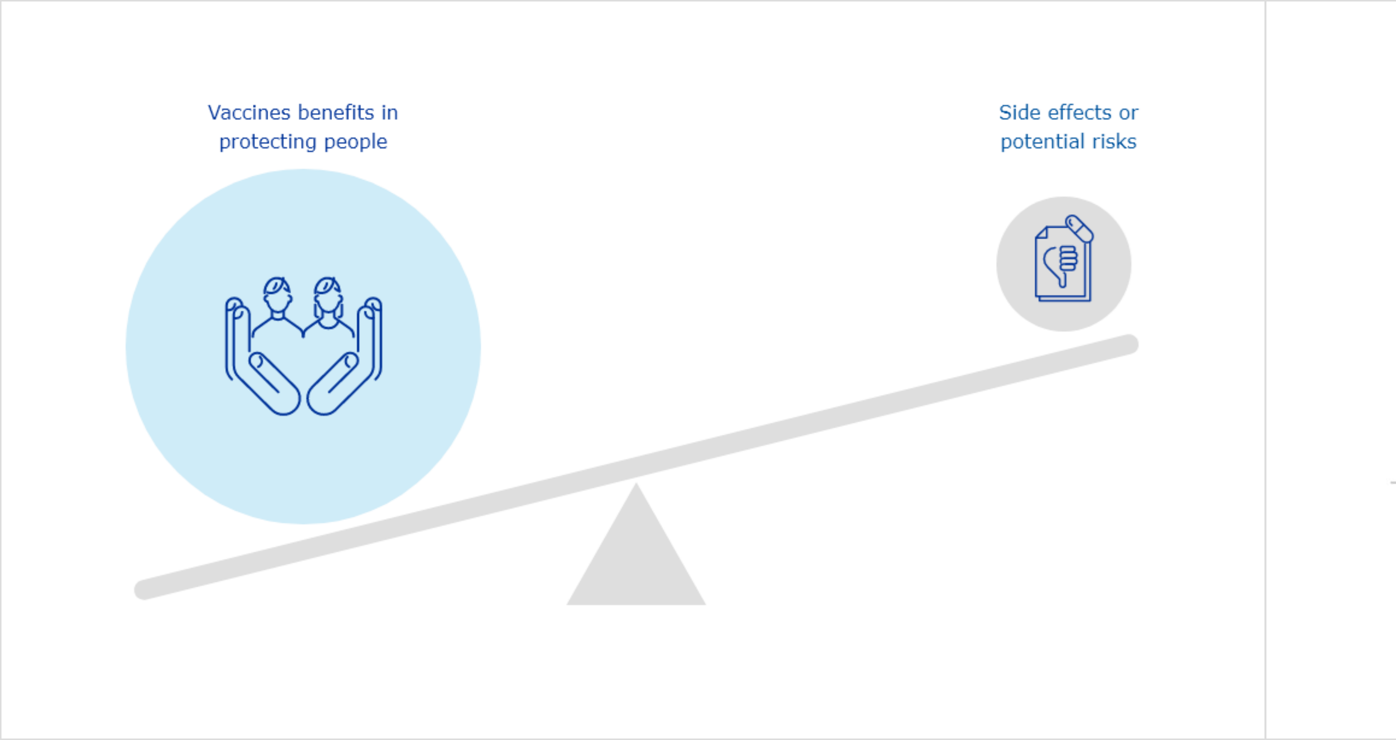

Q22. If answered yes, which of the figures do you prefer?

- A
- B
- C

If you have any suggestion of any of the figures presented in the documents, please describe below:

Q23. Do you think any information related to COVID-19 vaccines is missing? Please explain below

If you have suggestions or comments you want to make about any part of the documents, please describe below. The documents have numbered lines and pages, so it is easier to point to a specific part.

Thank you for your participation.
