# Supplementary material for: Stakeholders’ Understanding of European Medicine Agency’s COVID-19 Vaccine Information Materials in EU and Regional Contexts
Source: Vaccines (Basel). 2023 Oct 19;11(10):1616. doi: 10.3390/vaccines11101616 (PMC10610863; doi:10.3390/vaccines11101616)
Supplement: Supplementary file 1 [file vaccines-11-01616-s001.zip › Supplementary Material S3.pdf]

EUROPEAN MEDICINES AGENCY
SCIENCE MEDICINES HEALTH

Questionario sulla Valutazione delle Pagine Web EMA sullo sviluppo e le caratteristiche dei Vaccini COVID

1. **Lei è un (selezioni 1-2 opzioni):**
   - Popolazione generale
   - Membro di organizzazioni di pazienti
   - Membro di organizzazioni di operatori sanitari
   - Operatore sanitario individuale
   - Ex COVID-19 Positivo

   Se altro specifichi____

2. **Prima di leggere il materiale dell'EMA riguardante i vaccini contro COVID-19, quanto sarebbe disposto a vaccinarsi contro COVID-19, una volta che la vaccinazione sia disponibile e accessibile per lei?:** 1= molto improbabile; 7: molto probabile
3. **Dove cerca solitamente informazioni riguardanti i vaccini contro COVID-19? (Selezioni tutte le opzioni possibili)**
   - Il suo medico di famiglia/PLS
   - Farmacista di comunità
   - Infermiere
   - Leader di comunità
   - Agenzia Europea del farmaco (EMA)
   - Agenzia Italiana del farmaco (AIFA)
   - Riviste scientifiche
   - Ministero della Salute
   - La sua famiglia, colleghi e/o amici
   - Motori di ricerca (google, bing, ecc…)
   - Giornali (stampati o online)
   - Organizzazione Mondiale della Sanità (WHO)
   - Centro Europeo per la prevenzione e il controllo delle malattie (ECDC)
   - Social network (Facebook, instagram, twitter ecc…)
   - Altro
   - Non sa dove cercare

   Se altro specifichi ____

4. **L'inglese è la sua lingua?**
   - Si
   - No
5. **Se ha risposto di no, la lingua utilizzata rappresenta un ostacolo per la comprensione del documento?**
   - Si
   - No

6. **Come può l'EMA garantire che i vaccini contro COVID-19 siano sicuri ed efficaci (seleziona tutte le opzioni possibili)**
    - Applicando gli stessi standard elevati come per qualsiasi altro farmaco
    - Garantendo che tutti gli studi siano effettuati correttamente
    - Rivedendo i dati degli studi più velocemente ma con attenzione
    - Con il supporto di una task force di esperti dedicata
    - Soltanto gli studi su esseri umani volontari, eliminando gli studi sugli animali

7. **Vero o falso: i requisiti di sicurezza riguardanti i vaccini contro COVID-19 sono gli stessi a qualsiasi altro vaccino autorizzato in UE e non sono stati più bassi in periodo pandemico**
    - Vero
    - Falso
8. **Vero o falso: l'EMA effettua una valutazione scientifica riguardante la sicurezza, l'efficacia e la qualità del vaccino, prima di concludere che il vaccino possa essere approvato.**
    - Vero
    - Falso
9. **Una volta letto il documento "vaccini contro COVID-19: caratteristiche principali" (DOCUMENTO 1), ha trovato le informazioni chiare e utili?:** 1=confuse e per niente utili. 7=molto chiare e utili
10. **Potrebbe ora dare un punteggio alle seguenti categorie:** da 1 (per niente) a 7 (assolutamente), **in base alla sua esperienza con il documento?**
    - sono state fornite abbastanza informazioni per capire le caratteristiche principali
    - la lingua e i vocaboli utilizzati nel documento sono facili da comprendere
    - la lettura del documento ha incrementato le sue conoscenze riguardanti i vaccini contro COVID-19?
11. **Dopo aver letto "vaccini contro COVID-19: caratteristiche principali", quanto sarebbe disposto a vaccinarsi contro COVID-19 una volta che un vaccino sarà disponibile e sarà per lei accessibile?** 1= molto improbabile; 7: molto probabile
12. **Clicchi sul seguente link dove è stato pubblicato come pagina web il documento "vaccini contro COVID-19: caratteristiche principali":** https://www.ema.europa.eu/en/human-regulatory/overview/public-health-threats/coronavirus-disease-covid-19/treatements-vaccines/covid-19-vaccines-key-facts **Oltre a una pagina web, potrebbe esserle utile avere queste informazioni in un altro formato condivisibile?**
    - Si
    - No
    - Non lo so

    Se altro specifichi____

Il secondo documento: "vaccini contro COVID-19: sviluppo, valutazione, approvazione e monitoraggio" (DOCUMENTO 2) è un documento più dettagliato. Se non è un paziente o un operatore sanitario, la lettura del secondo documento e il completamento della seconda parte dell'indagine è facoltativa. Se lo ha letto, selezioni "Si" per continuare il questionario.

- Si
- No

13. **Quando viene monitorata la sicurezza dei vaccini?**
    - Solo durante le fasi 1-3 degli studi clinici sull'uomo
    - Solo quando il vaccino è approvato
    - Attraverso tutte le fasi di sviluppo clinico del vaccino, anche dopo l'approvazione e quando i vaccini sono utilizzati nella popolazione
    - Mai
14. **Cos'è una "rolling review"?**
    - Un processo di revisione rapido e rigoroso che l'EMA sta attualmente conducendo durante l'emergenza di sanità pubblica per una rapida valutazione dei medicinali contro COVID-19
    - Una revisione fatta dalle aziende farmaceutiche che hanno fatto i vaccini
    - Una revisione normalmente effettuata dall'EMA quando non ci sono pandemie in corso.
15. **Quali delle seguenti opzioni aiuta lo sviluppo dei vaccini contro COVID-19?**
    - Dialogo precoce tra sviluppatori ed esperti normativi
    - Associando più fasi della sperimentazione clinica quando non è compromessa la sicurezza
    - Utilizzando le conoscenze sulla sicurezza ed efficacia dei vaccini già disponibili sul mercato
    - Aumentando la capacità di produzione
    - Tutte le seguenti sono corrette
    - Nessuna delle seguenti è corretta
16. **Per quanto riguarda l'equilibrio rischio-beneficio sono vere le seguenti affermazioni. Quale dichiarazione pensa ribadisca con più chiarezza che i vaccini, come tutti farmaci, hanno benefici e rischi?**
    - Come ogni farmaco i vaccini hanno benefici e rischi, e le condizioni di autorizzazione all'immissione al commercio mira a massimizzare i benefici e minimizzare i rischi
    - Come ogni farmaco i vaccini hanno benefici e rischi, e nonostante l'alta efficacia nessun vaccino è efficace al 100% nel prevenire una malattia o nessun vaccino ha il 100% di sicurezza per tutte le persone vaccinate
17. **Dopo aver letto "vaccini contro COVID-19: sviluppo, valutazione, approvazione e monitoraggio" ha trovato le informazioni chiare e utili?:** 1=confuse e per niente utili. 7=molto chiare e utili
18. **Potrebbe dare un punteggio alle seguenti categorie** da 1 (per niente) a 7 (assolutamente) **in base alla sua esperienza con il documento?:**
    - sono state fornite abbastanza informazioni per capire i messaggi principali
    - i grafici aiutano a trasmettere i concetti principali
    - la lingua e i vocaboli usati nel documento sono semplici da capire
    - l'aver letto il documento ha incrementato le sue conoscenze sui vaccini contro COVID-19?

19. **Dopo aver letto "vaccini contro COVID-19: sviluppo, valutazione, approvazione e monitoraggio" sarebbe disposto a vaccinarsi contro COVID-19 una volta che diventi disponibile un vaccino?** 1=molto improbabile. 7=molto probabile
20. **Guardi cortesemente le due figure riportate sotto per mezzo minuto. Pensa che esse siano chiare e facili da comprendere?**

Resources figures

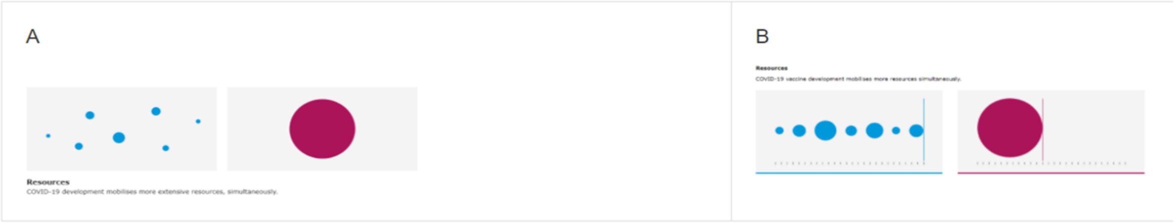

- Si
- No
- Non lo so

21. **Se ha risposto si, quale tra le due figure preferisce?**
    - A
    - B

Se ha qualche suggerimento riguardante le figure presentate nel documento lo scriva di seguito____

22. **Esamini le 3 figure per mezzo minuto. Pensa che esse siano chiare e facili di capire?**

Benefit-risk balance figures

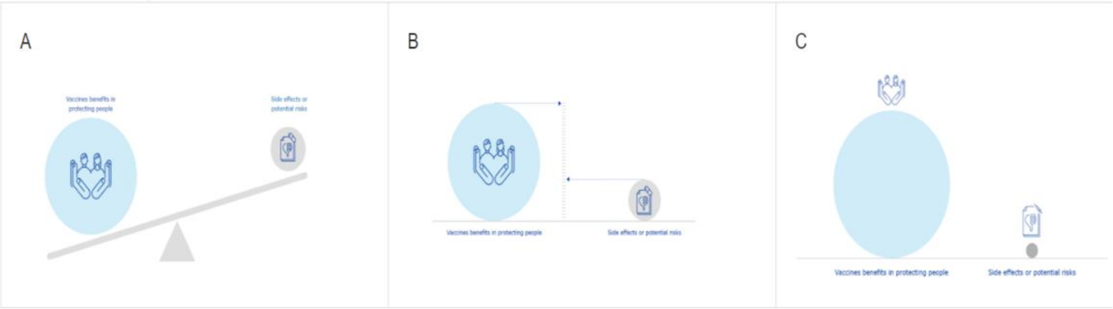

- Si
- No
- Non lo so

23. **Se ha risposto si, quale immagine preferisce?**
    - A
    - B
    - C

24. Se ha qualche suggerimento per qualche immagine presentata nel documento, lo scriva di seguito:

____

25. Pensa che manchi qualche informazione riguardante i vaccini contro COVID-19? Lo spieghi di seguito

____

Se ha suggerimenti o commenti riguardo qualche parte dei documenti, li scriva di seguito.
I documenti hanno linee e pagine numerate, quindi è più semplice identificare una parte specifica.

____
____
____
____
