# Supplementary material for: Stakeholders’ Understanding of European Medicine Agency’s COVID-19 Vaccine Information Materials in EU and Regional Contexts
Source: Vaccines (Basel). 2023 Oct 19;11(10):1616. doi: 10.3390/vaccines11101616 (PMC10610863; doi:10.3390/vaccines11101616)

Supplementary Figure 1. Usefulness of having key facts in another shareable material besides EMA webpage

EU-level (n=30) and regional (n=30) participants' interest in having "COVID-19 vaccines: key facts" in a shareable document.

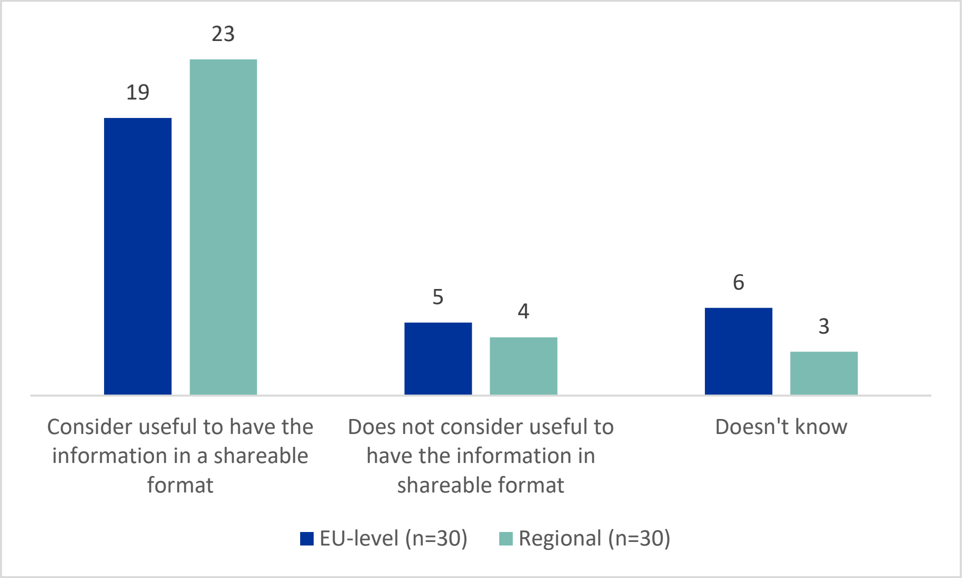

Supplement: Supplementary file 1 [file vaccines-11-01616-s001.zip › Supplementary Material S4.pdf]
